# Editorial: New developments in the intention-behavior gap for physical activity – recent trends, controversies, and a critical outlook

**DOI:** 10.3389/fpsyg.2023.1119973

**Published:** 2023-02-02

**Authors:** Chris Englert, Amanda Rebar, Ryan E. Rhodes, Ines Pfeffer

**Affiliations:** ^1^Department of Sports Sciences, Goethe University Frankfurt, Frankfurt, Germany; ^2^School of Health, Central Queensland University, Rockhampton, QLD, Australia; ^3^School of Exercise Science, Physical and Health Education, University of Victoria, Victoria, BC, Canada; ^4^Institute of Cognitive and Affective Neuroscience (ICAN), Medical School Hamburg, Hamburg, Germany

**Keywords:** physical activity, exercise, intention-behavior gap, effort, self-control, habit, executive function, implicit and explicit processes

Physical activity (PA) plays a predominant role in health promotion and disease prevention. Despite strong intentions, individuals often refrain from acting on intended behaviors due to various barriers (e.g., high stress, other priorities, and waning motivation), which is a phenomenon labeled as intention-behavior gap (e.g., Sheeran and Webb, 2016). Self-regulatory processes are particularly important to explain and bridge the intention-behavior gap as self-regulation enables us to modify our thoughts, feelings, moods, and impulses as well as other dominant response tendencies and to bring them in line with specific goals, aims, or norms (e.g., Rhodes and Yao, 2015; Finne et al., 2019; Pfeffer and Strobach, 2021; for a recent systematic review, see also Rhodes et al., 2022). Recent research has highlighted the importance of integrating explicit (e.g., motivational and self-regulatory processes) and implicit processes (e.g., habitual processes) to explain human behavior (e.g., Strobach et al., 2020; Rhodes, 2021). However, it is still unclear how explicit and implicit processes work together and, for example, if they operate in parallel (additive pattern) or may also interact (synergistically) in predicting PA behavior.

Therefore, the main aims of the current Research Topic were to expand our understanding of PA regulation, to identify the relevant processes affecting and bridging the intention-behavior gap, and to develop and evaluate treatments to promote and maintain PA behavior. In this context, dual-process theories (Hofmann et al., 2009; Brand and Ekkekakis, 2018; Strobach et al., 2020), action control theories (Rhodes and Yao, 2015), and integrated models of health behavior (Hagger and Hamilton, 2020) provide promising frameworks to examine and get new insights in the adoption and maintenance of PA behavior.

The review by Conner and Norman provides a theoretical discussion of the intention-behavior gap and the role of intention strength in the context of physical activity behavior. The authors provide insights in relevant intention-behavior moderators and they discuss features as well as predictors of intention strength. They conclude that stronger intentions predict more physical activity behavior and may be more stable over time, even though they are more difficult to change. As Conner and Norman explain, future research on the intention-behavior gap would benefit from a more systematic consideration of moderators of this relationship and an exploration of how these moderating effects might be explained.

The intensity of an exercise regime plays an important role for the subsequent health benefits as well as for future behavior. The study of Teixeira et al. examined how far the intensity of a training was in accordance with the preference and tolerance of participants and if this agreement/disagreement was associated with relevant predictors of future behavior (i.e., intention, habit) as well as behavior itself (i.e., exercise frequency). It was also hypothesized that agreement/disagreement interacted with physical activity enjoyment in predicting the dependent variables. Based on the results it seems to be important to tailor the intensity of exercise programs to the preference and tolerance of participants in order to foster future behavior.

To test the role of affective states for future physical activity behavior is a highly relevant (Rhodes and Gray, 2018). The study of Finne et al. aimed to examine the effect of affective states perceived directly after a training session on the intention to attend the next training as well as the attendance in the following week. In addition, the interaction effect between affective states and the intention to re-attend the course the following week was tested. In this longitudinal study over 13 weeks, affective states were positively related to intention, and intention mediated the effect of affective states on class attendance. In contrast to the assumption, affective states did not moderate the intention-class attendance relationship. Nevertheless, affective states during and after exercising should be further examined in relation to future behavior in the context of dual-process theories.

The study of Zhang et al. investigated the applicability of the planned behavior theory model (TPB-5) and TPB-6 model of enhanced physical exercise in college students, and explored the role of exercise commitment in the relationship between exercise intention and behavior. The results indicate that exercise commitment seems to be an important variable which might help to reduce the intention-behavior-gap.

More and Phillips in their study compared the utility of the Integrated Behavior Change Model (IBCM; Hagger and Chatzisarantis, 2014) — a social cognitive model that includes automatic factors involved in behavioral engagement and a moderator of the intention-behavior gap — to its theoretical predecessor, the Theory of Planned Behavior (TPB; Ajzen, 1991). While the IBCM was well-suited to explain intention formation, it did not predict significantly more variance than the TPB.

Text messages might be a cost-effective strategy for changing physical activity behavior in physically inactive individuals. The study of Tessier et al. examined the effects of goal-framing manipulation on physical activity attitude, perceived behavioral control, intention, goal content, as well as physical activity behavior. Low-active adolescents received messages targeting their salient beliefs with intrinsic vs. extrinsic-framing (compared to a control group). The results revealed that intrinsic goal framing compared to the extrinsic goal framing was not superior with regard to the behavior change process. Combining the message with a planning intervention was an effective strategy to promote physical activity behavior in low-active adolescents. However, the mechanisms by which these changes occurred could not be uncovered by the expected mediators. Further potential mechanisms should be examined in future studies.

Most efforts to change physical activity ideally translate from intentional to habitual regulation for long-term behavior change maintenance (Rhodes and Sui, 2021). Phillips and More aimed to identify the relevant factors which contribute to habit formation over a 4-week period, assuming that task complexity would play an important role. As postulated, intrinsic motivation, self-identity, and habit strength contributed to behavioral maintenance; however, only habit strength significantly predicted behavioral frequency over the 4-week period, independent of the respective task complexity. This is in line with theory that habit can underly long-term behavior change; but more is still needed to understand how initial intentions can translate into long-term physical activity.

Regular physical activity and the intention-behavior gap also plays an important role in the therapy of chronic diseases. The brief research report of Reicherzer et al. provides insights into perceived facilitators and barriers for physical activity of stroke patients with severe functional limitations. Based on semi-structured guided interviews, this qualitative study provided new insights into the intention-behavior gap in this specific population. The results might help to design tailored physical activity interventions. However, the small sample size of this study prevents the generalization of the results at this point in time as mobility impairments are a very heterogeneous within those with stroke diagnosis.

In recent years, dual process theories have become increasingly relevant for the examination of physical activity adoption and maintenance. The study of Zhu et al. examined the interplay of reflective (i.e., intention and action planning) and automatic processes (i.e., habit strength) in the prediction of exercise behavior in the volitional phase. Action planning is thought to make behavior more automatic by mentally pairing a situation with a specific exercise behavior (Gollwitzer, 1999). The authors take a closer look at the role of habit strength as a moderator of the planning-behavior relationship. As expected, action planning mediated the association between intention and behavior. Even though action planning might facilitate behavior because the plan is activated and executed automatically as soon as the anticipated situation arises, higher habit strength (i.e., automaticity) of exercise behavior seems to further strengthen this association. Therefore, action planning together with higher habit strength synergistically supported higher physical activity levels and diminished the intention-behavior gap rather than acting in a counterproductive manner.

## Author contributions

CE and IP wrote the first draft of the present manuscript. AR and RR contributed to its critical revision. All authors contributed to the article and approved the submitted version.
